# The mechanism of palmatine-mediated intestinal flora and host metabolism intervention in OA-OP comorbidity rats

**DOI:** 10.3389/fmed.2023.1153360

**Published:** 2023-04-20

**Authors:** Lishi Jie, Zhenyuan Ma, Yifan Gao, Xiaoqing Shi, Likai Yu, Jun Mao, Peimin Wang

**Affiliations:** ^1^Department of Orthopaedics and Traumatology, Jiangsu Provincial Hospital of Traditional Chinese Medicine, The Affiliated Hospital of Nanjing University of Chinese Medicine, Nanjing, China; ^2^Key Laboratory for Metabolic Diseases in Chinese Medicine, First College of Clinical Medicine, Jiangsu Provincial Hospital of Traditional Chinese Medicine, Nanjing University of Chinese Medicine, Nanjing, China

**Keywords:** osteoarthritis, osteoporosis, palmatine, gut microbiota, metabonomics

## Abstract

**Background:**

ErXian decoction is a Chinese herbal compound that can prevent and control the course of osteoarthritis (OA) and osteoporosis (OP). OP and OA are two age-related diseases that often coexist in elderly individuals, and both are associated with dysregulation of the gut microbiome. In the initial study, Palmatine (PAL) was obtained by liquid chromatography-tandem mass spectrometry (LC-MS/MS) and network pharmacological screening techniques, followed by 16S rRNA sequencing and serum metabolomics of intestinal contents, to explore the mechanism of PAL in the treatment of OA and OP.

**Methods:**

The rats selected for this study were randomly divided into three groups: a sham group, an OA-OP group and a PAL group. The sham group was intragastrically administered normal saline solution, and the PLA group was treated with PAL for 56 days. Through microcomputed tomography (micro-CT), ELISA, 16S rRNA gene sequencing and non-targeted metabonomics research, we explored the potential mechanism of intestinal microbiota and serum metabolites in PAL treatment of OA-OP rats.

**Results:**

Palmatine significantly repair bone microarchitecture of rat femur in OA-OP rats and improved cartilage damage. The analysis of intestinal microflora showed that PAL could also improve the intestinal microflora disorder of OA-OP rats. For example, the abundance of Firmicutes, Bacteroidota, Actinobacteria, Lactobacillus, unclassified_f_Lachnospiraceae, norank_f_Muribaculaceae, Lactobacillaceae, Lachnospiraceae and Muribaculaceae increased after PAL intervention. In addition, the results of metabolomics data analysis showed that PAL also change the metabolic status of OA-OP rats. After PAL intervention, metabolites such as 5-methoxytryptophol, 2-methoxy acetaminophen sulfate, beta-tyrosine, indole-3-carboxylic acid-O-sulfate and cyclodopa glucoside increased. Association analysis of metabolomics and gut microbiota (GM) showed that the communication of multiple flora and different metabolites played an important role in OP and OA.

**Conclusion:**

Palmatine can improve cartilage degeneration and bone loss in OA-OP rats. The evidence we provided supports the idea that PAL improves OA-OP by altering GM and serum metabolites. In addition, the application of GM and serum metabolomics correlation analysis provides a new strategy for uncovering the mechanism of herbal treatment for bone diseases.

## 1. Introduction

Osteoarthritis (OA) and osteoporosis (OP) have gradually become an urgent global public health problem and a large number of middle-aged and elderly people, an important component of the burden of social medical costs (1, 2). According to previous studies, OA would become the fourth global pandemic by 2020 (3). Furthermore, OP is a very common disease that affects 200 million people worldwide (4). Importantly, OA and OP are two kinds of bone diseases that are closely related and have the noteworthy feature of abnormal reconstruction of subchondral bone (5). Although clinically OP and OA have different pathological characteristics, their risk factors are similar and closely related and include aging, metabolic changes and inflammation (6). Therefore, both diseases are described as age-related. Due to the high prevalence of OA and OP and their heavy burden on patients and the function of social medical services, OA and OP have gradually become an ongoing focus in current scientific research (7). Studies have shown that both OA and OP are closely related to the equilibrium state of the gut microenvironment, suggesting a potentially important factor imbalance in the gut microbiota (8). An increasing number of human and animal studies, especially in recent years, have shown the existence of the intestinal bone axis and have revealed that there are some correlations between the transgenes and their metabolites and the pathogenesis of OA and OP, which may be a potential target for intervention (9).

There is increasing evidence that there is an inextricably linked relationship between the gut microbiome (GM) and bone homeostasis involving host microbiome crosstalk (8). The GM is a very diverse ecosystem consisting of 10 to 100 trillion microorganisms and plays an important role in the body’s metabolic system. The effects of the intestinal flora on OP and OA share many common mechanisms, including influencing nutrient absorption, changing hormone levels, altering the intestinal mucosal barrier and mediating immunity (10–13). Studies on OP have shown that the consumption of specific metabolites, such as Glucagon-like peptide-1 and peptide YY, by gut microbes affects the endocrine functioning of the host, and these metabolites play a regulatory role in OP processes, regulates the differentiation of bone marrow Mesenchymal stem cell between bone cells and adipocytes (14, 15). In OA, inflammatory status, obesity, metabolic syndrome and intestinal flora disorder are strongly correlated and closely related to OA risk. For example, disruption of the GM may slow OA by reducing inflammatory states and reducing the expression of Wnt signaling regulatory proteins (16). People pay more and more attention to the disease of bone metabolism from the perspective of intestinal flora. GM affects joints by regulating inflammation and metabolism, but GM alone does not fully explain the phenomenon. Thus, the combined application of omics techniques, such as metabolomics and 16S RNA sequencing, to explore this complex problem becomes particularly important.

Traditional Chinese medicine decoction ErXian decoction (EXD) was included in the famous Ming Dynasty medical book Wenbing Tiaobian (17). In clinic, erxian decoction has a good curative effect (18). Our previous research results showed that joint disease from both OA and OP produced more severe cartilage damage than that from OA alone. Notably, EXD can significantly improve cartilage damage and reverse the protein expression of SOX9, COL2A1, and COMP. In addition, This benign intervention involves cysteine, deoxycholate, and D-turanose, as well as their associated glycolysis/gluconeogenesis, pantothenic acid, and CoA biosynthesis (19). In this study, in combination with network pharmacology, we screened the possible monomer components of EXD in the treatment of OP and OA with palmatine (PAL). PAL has been found to have unique pharmacological effects, including anticancer, antioxidant, anti-inflammatory, antibacterial, antiviral and lipid-modulating effects (20). It has been shown that PAL can decrease the differentiation of osteoclasts by inhibiting the expression of RANKL and OPG in osteoblasts (21). In addition, In the treatment of OA, PAL can improve the pathological progression of OA by inhibiting Wnt/β-catenin and Hedgehog signaling pathway (22). Therefore, PAL may be an effective drug for the treatment of OA and OP. In this study, we used a variety of technical approaches to explore the potential mechanisms of Pal in the treatment of OA and OP. Moreover, 16S rRNA gene sequencing and metabolomics techniques were used to study the mechanism of PAL treatment of OA and OP from the perspective of intestinal flora and host metabolism.

## 2. Materials and methods

### 2.1. Screening of potential components of EXD in the treatment of OA and OP

#### 2.1.1. Construction of the drug activity component library

The Traditional Chinese Medicine Systems Pharmacology Database and Analysis Platform (TCMSP)^1^ and the Bioinformatics Analysis Tool for Molecular mechANism of Traditional Chinese Medicine (BATMAN-TCM)^2^ databases were used to retrieve the active ingredients of EXD. The chemical composition information of EXD were retrieved from TCMSP. A screening condition of 30% oral bioavailability (OB) and 0.18 drug similarity (DL) was established to achieve the active ingredients. The BATMAN-TCM procedure set the similarity score between drug and target as score cutoff + 20 and adjusted *P*-value 0.05.

#### 2.1.2. Disease target data set

Search and filter relevant gene targets using the keyword “Osteoarthritis” and “Osteoporosis” on GeneCard,^3^ DisGeNET,^4^ OMIM,^5^ DrugBank,^6^ and PharmGKB^7^ databases. Using Venn diagrams, drugs and disease targets were shown logically. Drug targets were mapped to disease targets, and the common targets of EXD for OA and OP were screened.

#### 2.1.3. PPI network and core target analysis

STRING database was used to analyze the protein interaction networks based on the common targets obtained, and a protein-protein interaction diagram (PPI) was created. Multiple proteins, Homo sapiens, with a confidence level of 0.7 were the conditions. The “CytoNCA” plug-in in Cytoscape software was used for topology attribute analysis to obtain the topology parameters of betweenness centrality (BC), closeness centrality (CC), degree centrality (DC), eigenvector centrality (EC), network centrality (NC), and local average connectivity (LAC). To form the core PPI network, nodes with BC, CC, DC, EC, NC, and LAC greater than the median were screened out.

#### 2.1.4. Networking of drugs and diseases

A target network for EXD treatment for OA and OP was constructed using Cytoscape (3.7.2). For the visual analysis of the target network, each node represents the relevant target of EXD and OA-OP, and the edge represents the interaction between these biological analyses.

### 2.2. Experimental verification

#### 2.2.1. Drugs and reagents

Palmatine (PAL, purity >98%, HY-N0110A) was provided by MCE (NJ, USA); Osteopontin (OPN) Antibody (#AF0227)‵ MMP13 Antibody (#AF5355)‵MMP3 Antibody (#AF0217) was provided by Affinity Biosciences (Jiangsu, China). RUNX2 Antibody (sc-390351) was provided by Santa Cruz Biotechnology (CA, USA). The enzyme-linked biotechnology company (Shanghai, China) provided Rat Estradiol (E2) ELISA Kit (YJ002871),Rat Bone gla protein; Osteocalcin (BGP; OCN) ELISA Kit (YJ420711),Rat alkaline phosphatase (ALP) ELISA Kit (YJ003360), Rat 1,25-dihydroxyvitamin D3 (1,25(OH)_2_D_3_) ELISA Kit (YJ403912),Rat phosphorus (P) ELISA Kit (YJ103904) and Rat calcium (Ca) ELISA Kit (YJ103924).

#### 2.2.2. Instruments

Multifunctional microplate reader (Enspire, Perkin Elmer, USA); Skyscan 1276 Micro-CT Imaging System (Skyscan, Kontich, Belgium); triple Tof5600 triple quadrupole mass spectrometry, AB SCIEX; ExionLC AD liquid chromatography System, AB SCIEX; HSS T 3 column (100 mm × 2.1 mm I.D., 1.8 M M), waters, USA; Abi Geneamp 9700 PCR machine (ABI, USA); the Milli-Q ultra-pure water system (Millipore, Billerica, MA, USA); centrifuge 5702 low-speed centrifuge (Eppendorf, Germany); Leica DMI8 fluorescence inversion microscope (Leica, Germany).

#### 2.2.3. Animal experiment and sample collection

Under the approval of the Animal Ethics Committee of Nanjing University of Chinese Medicine (202209A034), female Sprague Dawley (SD) rats (6 to 8 weeks of age) were purchased from GemPharmatech Co., Ltd. The animals were raised in a controlled temperature, humidity and 12 h/dark environment. As previously reported, the OA-OP rat model was established through the combination of anterior cruciate ligament resection (ACLT) and ovariectomy (OVX) (19). Two week after recovery from surgery, Three groups of rats were assigned randomly: blank (*n* = 6), OA-OP + PAL (*n* = 6) and OA-OP (*n* = 6) (23). In the PAL group, PAL (100 mg kg^–1^) was administered by gavage once a day for 56 days (24, 25). All rats were sacrificed on the 56th day to obtain femurs, tibias and blood. The rats were first weighed and anesthetized with a 3% sodium pentobarbital solution at a dose of 100 mg/kg according to body weight, and then the rats were euthanized by treatment of cervical dislocation. The collected blood samples were coagulated and centrifuged (2000 rpm, 10 min, 4°C) to obtain serum.

#### 2.2.4. Microcomputed tomography

The lower limbs of rats were fixed with 4% paraformaldehyde for 24 h and then soaked in PBS solution until used. We performed Micro-CT scans using the Skyscan 1276 Micro-CT system (Skyscan, Kontich, Belgium). Bone trabecular volume fraction (BV/TV,%), trabecular number (TB. N,/MM), trabecular thickness (TB. Th, MM) and trabecular space (TB. SP, MM) were calculated.

#### 2.2.5. ELISA Assay

According to the manufacturer’s instructions, Ca, P, 1,25(OH)_2_D_3_, E_2_, BGP, OCN, and ALP were determined in rat serum with ELISA kits.

#### 2.2.6. Testing and evaluation of histopathology

After the intervention, the rats were euthanized and the samples of knee joints were collected. Fixed with 4% paraformaldehyde for 24 h. Decalcification was carried out with 10% EDTA solution. The sections were made after paraffin embedding. H & E, Lycopene O/rapid green and TRAP staining were performed. After staining was completed, image acquisition was performed using an upright light microscope (Eclipse E100, Nikon, Japan) and an imaging system (DS-U3, Nikon, Japan).

#### 2.2.7. Measurement of serum metabolites using LC–MS/MS

##### 2.2.7.1. Metabolite extraction

A liquid sample of methanol: acetonitrile (1:1, V/V) solution was used. The mixture was then sonicated at 5°C at 40 khz. The sample was placed at −20°C to precipitate the protein. The supernatant was evaporated and dried under the flow of nitrogen after centrifugation at 4°C for 10 min at 12000 × *g*. For UHPLC-MS/MS analysis, samples were reconstructed in a loading solution (1:1, V/V) of acetonitrile: water by transient sonication in a 5°C water bath. The extracted metabolites were centrifuged at 4°C, 12000 × *g* for 1 min, and the cleared supernatant was transferred to sample vials for LC-MS/MS analysis.

##### 2.2.7.2. UPLC–MS/MS analysis

###### 2.2.7.2.1. Chromatographic conditions

The samples were separated on HSS T 3 column before entering into mass spectrometry. Using 0.1% formic acid in water: acetonitrile (95:5, V/V) as mobile phase A, 0.1% formic acid in acetonitrile: Isopropanol: water (47.5:47.5, V/V) as mobile phase B, the volume of each sample was 10 μl and the analysis time was set at 5 min, the flow rate of 0.4 mL/min and the column temperature of 40°C were maintained at the same time.

###### 2.2.7.2.2. MS conditions

The UPLC system was designed under the following conditions: the source temperature was 550°C, the curtain gas (CUR) was 30 psi, the ion source GAS1 and Gas2 were 50 psi, the ion spray voltage floating (ISVF) was −4000 V, the positive mode was 5000 V, the potential of de-clustering was 80 V, and the voltage of ion spray was −4000 V The collision energy (CE) of 20–60e V rolling MS/MS data acquisition was adopted in the information dependent acquisition (Ida) mode. Samples were tested in the range of 50–1000 m/z.

##### 2.2.7.3. Data processing and annotation

liquid chromatography-tandem mass spectrometry data were preprocessed by Progenesis QI (Waters Corporation, Milford, CT, USA) following mass spectrometry detection, and a three-dimensional data matrix in comma separated value (CSV) format was exported. At the same time, the metabolites were searched and identified in the Human Metabolome Database (HMDB), Metlin MassBank and MzCloud databases.

Perform variance analysis was conducted on the matrix file after data preprocessing. The R package ropls (Version 1.6.2) was used to perform orthogonal least partial squares discriminant analysis (OPLS-DA) and 7-cycle interactive validation was used to evaluate the stability of the model. The selection of significantly different metabolites was determined based on the variable importance in the projection (VIP) obtained by the OPLS-DA model and the *p*-value of Student’s *t*-test, and the metabolites with VIP > 1 and *p* < 0.05 were significantly different metabolites. Differential metabolites among the two groups were summarized and mapped into their biochemical pathways through metabolic enrichment and pathway analysis based on a Kyoto Encyclopedia of Genes and Genomes (KEGG) database search.

#### 2.2.8. 16S RNA sequencing

##### 2.2.8.1. DNA extraction

A soil DNA kit (Omega Bio Tek, Norcross, GA, USA) was used to extract the total genomic DNA from the microbial community.

##### 2.2.8.2. Library preparation and sequencing

Samples meeting quality control criteria were subjected to amplification of the V3-V4 region of the 16S rRNA gene by Abi GeneAmp 9700 PCR thermal cycler (ABI, CA, USA) using primers from 338F (5′-ACTCCTACGGGAGGCAGCAG-3′) and 806R (5′-GGACTACHVGGGTWTCTAAT-3′), Purified amplicons were pooled in equimolar amounts and were paired-end sequenced on an Illumina MiSeq PE300 platform/NovaSeq PE250 platform (Illumina, San Diego, CA, USA). Raw sequencing reads were deposited in NCBI’s Sequence Read Archive (Accession Number: SUB12688536).

##### 2.2.8.3. Analysis of sequencing data

Similarity of microbial communities between samples was determined by principal coordinate analysis (PCOA) based on Bray-curtis dissimilarity, using the vegan v2.5-3 package. Permutation multivariate analysis of variance (Permanova) test was used to assess the percentage of variance explained by treatment of vegan v2.5-3 packages and its statistical significance. According to the LEfSe linear discriminant analysis analysis, there were significantly abundant taxa (phylum genus) in different groups of bacteria (LDA score >2, *p* < 0.05). Species were selected for correlation network plot analysis according to Spearman correlation | r | > 0.6 *p* < 0.05.

#### 2.2.9. Western blotting

Rat cartilage and femur were ground in liquid nitrogen and homogenized with Ripa buffer. The protein concentration was determined by BCA method. Proteins were first separated using 10% PAGE and then transferred to PVDF membranes. PVDF membranes were blocked with TBST buffer containing 5% skim milk powder for 1 h at room temperature and then rinsed with TBST. After incubation with the first antibody at 4°C overnight, the membrane was incubated with the corresponding second antibody for 1 h. Protein bands were displayed using chemiluminescent reagents, and ImageJ was used to quantify protein intensity.

#### 2.2.10. Statistical analysis

SPSS software 22.0 and GraphPad Prism 8.0 were used for data analysis. The results are expressed as the mean ± standard deviation (x ± s). Group comparisons were assessed with one-way ANOVA, and *p* < 0.05 was regarded as statistically significant.

## 3. Results

### 3.1. Screening of active ingredients of drugs

**FIGURE 1:**
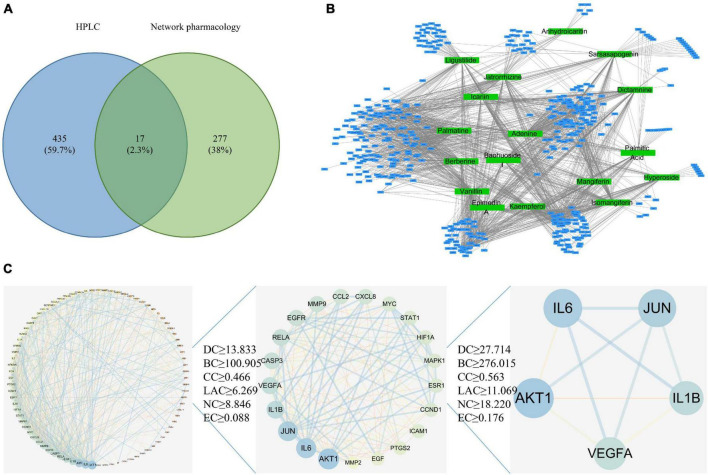
Network pharmacology analysis. **(A)** Common target acquisition. **(B)** Drug-disease target network construction. The blue circles represent common genes, and the green circles represent active ingredients of EXD. **(C)** PPI network diagram.

According to the screening conditions, 317 kinds of effective ingredients from EXD were obtained. After deleting invalid and repeated targets, 294 active ingredients were included in this analysis. According to our previous research, 452 monomer components were obtained by HPLC analysis. The network pharmacology screening results and the identification results of HPLC were mapped, and 17 common components were obtained, as shown in Table 1 and Figure 1A.

**TABLE 1:** A total of 17 common active components.

Name	Formula	Class	MZMED
Palmitic acid	C16H32O2	Fatty acyls	255.2325528
Vanillin	C8H8O3	Phenols	153.0544604
Dictamnine	C12H9NO2	Alkaloids	200.0706866
Adenine	C5H5N5	Alkaloids	136.0618078
Ligustilide	C12H14O2	Dihydrofurans	191.1068483
Palmatine	C21H22NO4 +	Alkaloids	352.1546634
Jatrorrhizine	C20H20NO4 +	Alkaloids	338.1387217
Berberine	C20H18NO4 +	Alkaloids	336.1233456
Baohuoside I	C27H30O10	Flavonoids	515.1902982
Kaempferol	C15H10O6	Flavonoids	287.0548828
Epimedin A	C39H50O20	Flavonoids	839.289447
Icariin	C33H40O15	Flavonoids	677.2423439
Hyperoside	C21H20O12	Flavonoids	465.1021701
Isomangiferin	C19H18O11	Xanthones	423.0916829
Mangiferin	C19H18O11	Xanthones	423.0919346
Sarsasapogenin	C27H44O3	Terpenoids	417.3354335
Anhydroicaritin	C21H20O6	Flavonoids	369.1328378

Based on 17 common components and the GeneCard, DisGeNET, OMIM, DrugBank, and PharmGKB databases, 447 drug targets were screened. A network diagram of the common targets of the drug active ingredients was constructed by using Cytoscape software and included 464 points and 1677 edges (Figures 1B, C). With the degree value as the main reference for topological analysis, the top five active ingredients were palmatine, adenine, vanillin, kaempferol, and isomangiferin. These top drug ingredients may be the key compounds of EXD in the treatment of OA and OP. According to the topological analysis results and previous studies (Supplementary Table 1), we chose PAL to carry out follow-up experiments (19). A detailed procedure how palmatine was identified was illustrated in the flow chart (Supplementary File 1).

### 3.2. Palmatine improves the bone structure of rats

**FIGURE 2:**
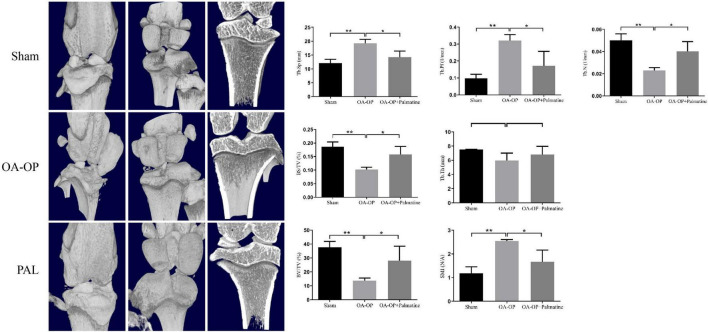
Effect of PAL on bone trabecular morphometric parameters in OA-OP rats. Micro-CT images of the distal femur and proximal tibia 56 days after PAL administration; Tb. N, BV/TV, BS/TV, Tb. Th, Tb. Pf, Tb. Sp, and SMI, 56 days after PAL administration Micro-CT analysis. *Statistically significant difference (*P* < 0.05). **Statistically significant difference (*P* < 0.01).

Micro-CT has become a key tool for evaluating bone microstructure in animal models of OP (26). The isolated femur samples from each group were scanned with micro-CT, and the area of interest was reconstructed in three dimensions (Figure 2). The results showed that compared with the sham group, the OA-OP group trabecular bone was reduced and broken, and the distance between the trabecular bone was widened, indicating that the bone microstructure in the OA-OP group was destroyed, and that bone loss and osteoporosis occurred. However, after PAL intervention, it was observed that the number of bone trabecule increased, the distance between bone trabecule decreased, the thickness of bone trabecule increased, and the bone strength partially recovered, preventing the process of bone loss. In addition, compared with the sham group, femur Tb. N, BV/TV, BS/TV, and Tb. Th in the model group were significantly decreased (*p* < 0.05), while Tb. Pf, Tb. Sp, and SMI were significantly increased (*p* < 0.05). Tb. Pf and SMI can measure the degree of rod-like and plate-like bone trabecula, and their increase indicates the change in bone trabecula from plate-like to rod-like. Combined with the decrease in bone volume and bone trabecular thickness and number, the rat OVX model was successfully established. Compared with the OA-OP group, Tb. N, BV/TV, BS/TV, and Tb. Th of the femur in the PAL group was significantly increased (*p* < 0.05), Tb. Pf and SMI were significantly decreased (*p* < 0.05).

### 3.3. Effect of palmatine on bone metabolism in rats

**FIGURE 3:**
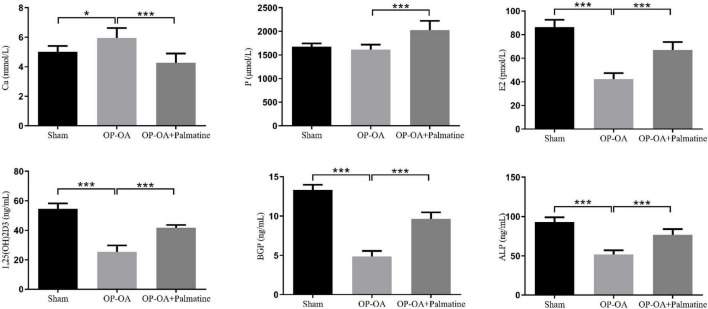
Effect of PAL on bone metabolism in OA-OP rats. Quantitative analysis of serum levels of E2, ALP, BGP, 1,25(OH)2D3, Ca, and P. *Statistically significant difference (*P* < 0.05). **Statistically significant difference (*P* < 0.01). ***Statistically significant difference (*P* < 0.001).

The results show (Figure 3), compared with the sham group, the OA-OP group serum E2, ALP, BGP, and 1,25(OH)_2_D_3_ contents decreased, while the Ca content increased (*p* < 0.05). The levels of E2, ALP, BGP, and 1,25(OH)_2_D_3_ in serum of OA-OP rats were increased in PAL group (*p* < 0.05).

### 3.4. Palmatine improved OA and OP processes in rats

**FIGURE 4:**
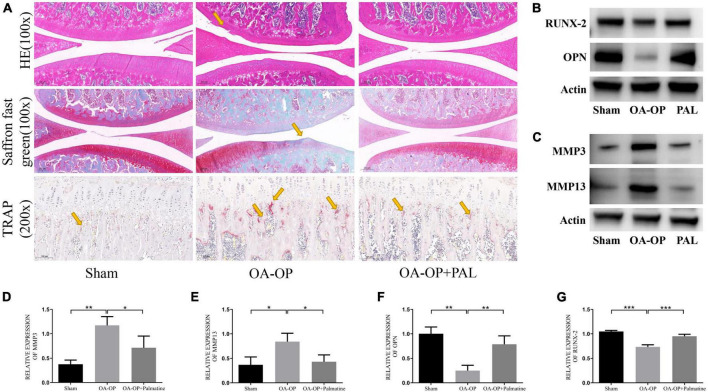
Effect of PAL on bone pathological section of Op Oa Rats. **(A)** Representative pictures of rat knee tissue sections stained with H & E, Saffron O/Fast Green and TRAP. **(B)** Representative images of protein bands in rat tissues. **(C)** Representative images of protein bands in rat cartilage. **(D)** The relative expression levels of MMP3 in cartilage. **(E)** The relative expression levels of MMP13 in cartilage. **(F)** The relative expression levels of OPN in femur. **(G)** The relative expression levels of RUNX-2 in femur. *Statistically significant difference (*P* < 0.05). **Statistically significant difference (*P* < 0.01). ***Statistically significant difference (*P* < 0.001).

Compared with the OA-OP group, the sham group cartilage surface was smooth, while the PAL group showed less cartilage destruction. The OARSI score was consistent with that of solid green staining, and OA-OP group’s score was significantly higher than SHAM group’s, the OARSI score of the PAL group was lower than that of the OA-OP group. Compared with the sham group, in the OA-OP group, H & E staining of femur showed that PAL could ameliorate the destruction of extra-articular matrix induced by OA-OP. In TRAP staining, the number of positive cells in SHAM group was less than that in OA-OP group, while PAL reduced the number of positive cells in OA-OP group. The results of WB (Figure 4) showed that the protein expression levels of MMP3 and MMP13 in the cartilage of the OA-OP group were increased (*p* < 0.05), while PAL could decrease the protein expression levels of MMP3 and MMP13 in the cartilage of the KOA group (*p* < 0.05). In the OA-OP group, the protein expression levels of Runx-2 and OPN were decreased (*p* < 0.05), whereas PAL could increase the protein expression levels of RUNX-2 and OPN in KOA cartilage (*p* < 0.05).

### 3.5. PAL effects on OA-OP rats’ intestinal flora composition

**FIGURE 5:**
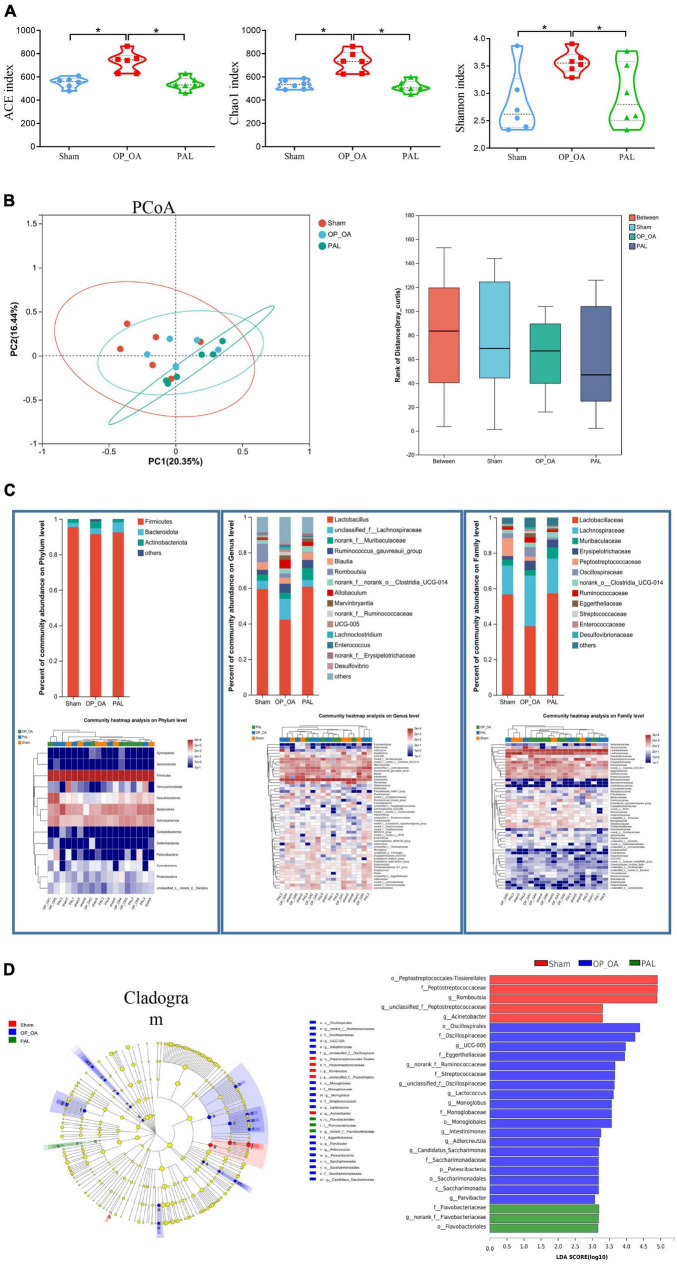
Intestinal flora analysis. **(A)** The alpha diversity of intestinal microorganisms was evaluated through Chao1, ACE, and Shannon; **(B)** pCoA scoring chart and box diagram of distance between groups; **(C)** species composition abundance map and abundance clustering heatmap of phylum, genus and family level; **(D)** intestinal microbial LEfSe from domain to species and LDA showed scores of these specific bacteria. *Statistically significant difference (*P* < 0.05).

To further study the effect of PAL on intestinal microflora, fecal microflora of 18 samples from 3 groups were analyzed by 16S RNA sequencing. For the α-diversity, there were significant differences in the ACE, Chao1 and Shannon indexes among the three groups (Figure 5A), suggesting that PAL treatment may have a significant effect on the intestinal microflora of OA-OP rats. Using the PCoA method, we analyzed β-diversity. As shown in Figure 5B, PCoA showed a significant clustering of microbiota composition in each group and showed that the microbiota community of rats in the OA-OP group was significantly different from that in the sham and PAL groups, suggesting that PAL treatment may improve OA and OP in rats by regulating GM imbalance. Next, to assess specific changes in GM, the relative abundance of dominant groups was further analyzed (Figure 5C). At the phylum level, Firmicutes, Bacteroidota and Actinobacteriota were predominant. At the genus level, Lactobacillus, unclassified_f_Lachnospiraceae and norank_f_Muribaculaceae were predominant. At the family level, Lactobacillaceae, Lachnospiraceae and Muribaculaceae were predominant. To further explore the differences in intestinal microbiota among the sham, OA-OP and PAL groups, we used LEfSe to identify specific changed bacterial phenotypes at each phylogenetic level. As shown in Figure 5D, *p* < 0.05 and LDA > 3.0 were biomarkers of significant differences in the screening rank sum test. A total of 27 specific bacteria were divided into 3 groups, including 5 specific bacteria in the sham group, 19 in the OA-OP group and 3 in the PAL group.

### 3.6. Effect of PAL on host metabolism in OA-OP rats

**FIGURE 6:**
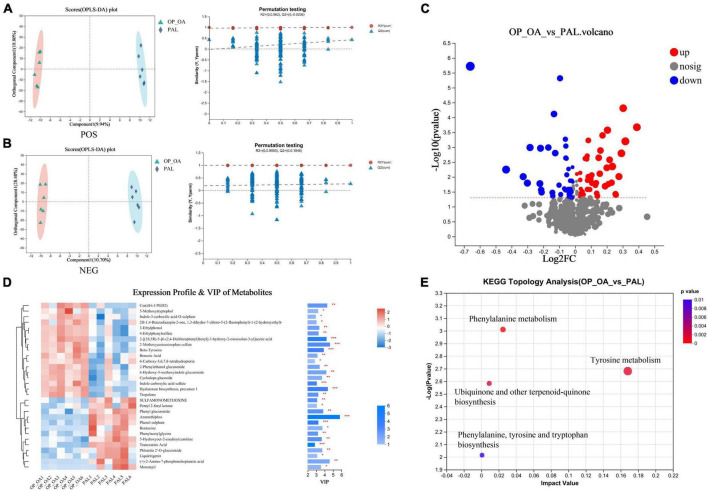
Metabonomic analysis. **(A)** OPLS-DA score plot and OPLS-DA permutation test chart (POS); **(B)** OPLS-DA score plot and OPLS-DA permutation test chart (NEG); **(C)** comparison between OA-OP and PAL single-dimensional metabolite volcano map. Threshold setting *p* < 0.05 and | log2fc | ≥ 1 (FC, fold change); **(D)** VIP analysis chart. The left side is the metabolite cluster tree, and the color represents the relative expression amount of this metabolite in this group of samples. The right side is a VIP bar graph of metabolites. The bar length indicates the contribution value of the metabolite to the difference between the two groups. The larger the value, the greater the difference between the two groups. The bar color indicates the significant difference in metabolites between the two groups of samples. **(E)** Metabolic pathway analysis of plasma differential metabolites.

Blood samples were analyzed by LC-ESI-MS/MS system to study the metabolic status of each group of hosts. PLS-DA was used to assessed differences in blood metabolic profiles between the two groups. The PAL group was significantly separated from the OA-OP group, indicating that PAL could regulate the metabolism of model rats (Figures 6A, B). The PAL group was significantly separated from the OA-OP group, indicating that PAL could regulate the metabolism of model rats. In the next step, potential biomarkers were screened between the sham and OA-OP groups based on OPLS-DA. Moreover, the PLS-DA model was verified by a displacement test, which showed that the fit was good. The online HMDB, Metlin, MassBank, MzCloud, and databases were used to screen 93 potential biomarkers, including 46 upregulated and 47 downregulated metabolites (Figure 6C and Supplementary Table 2). We performed a data clustering analysis of the top 30 metabolites by VIP value to show differences in metabolite expression, (Figure 6D). The horizontal coordinate indicates the sample name, and the vertical coordinate indicates the top 30 differential metabolites according to VIP value. In the figure, red represents upregulation, and green represents downregulation of metabolites. The results showed that there were significant differences in the levels of metabolites between the groups. Furthermore, the metabolic pathways of 93 different metabolites were analyzed to explore the metabolic pathways regulated by PAL. When the *p* < 0.05, the metabolic pathway was considered to be significantly correlated with PAL intervention. These pathways are mainly involved in tyrosine metabolism, phenylalanine metabolism, ubiquinone and other terpenoid-quinone biosynthesis and so on (Figure 6E and Supplementary Table 3).

### 3.7. Correlation analysis between metabonomics and GM

**FIGURE 7:**
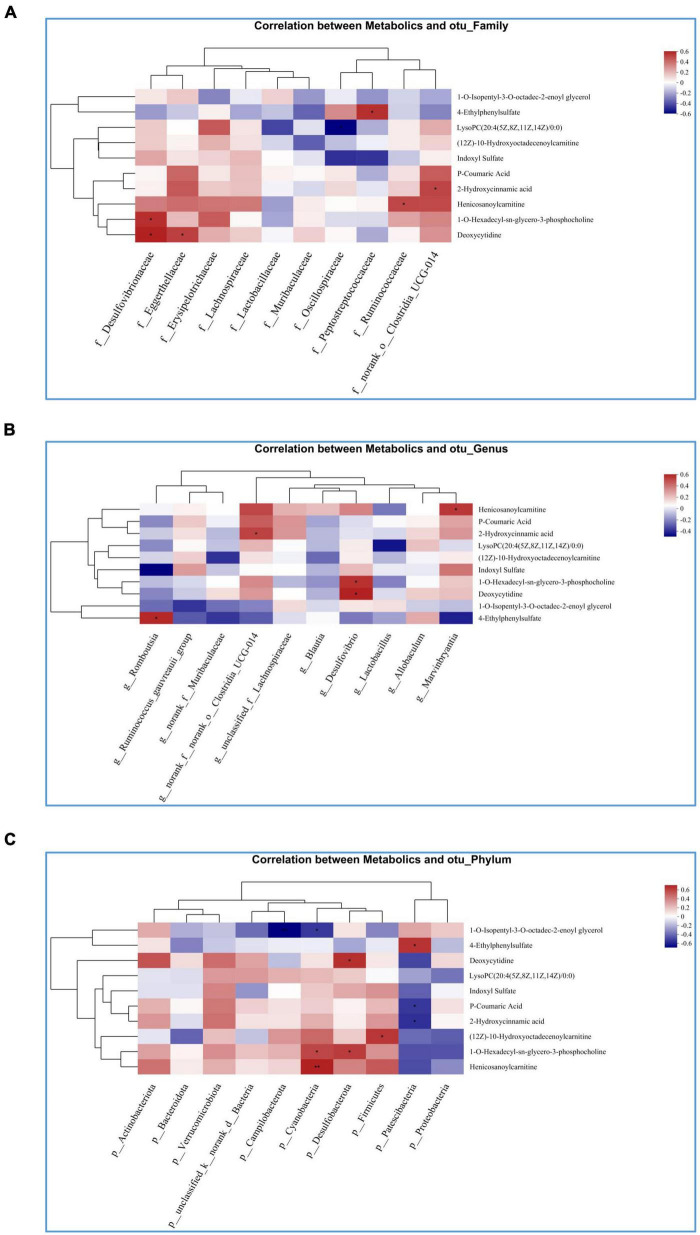
Heatmap of the correlation analysis between gut microbiota and targeted metabolic profiling. **(A)** Correlation between intestinal flora and metabolites at the family level; **(B)** correlation between intestinal flora and metabolites at genus level; **(C)** correlation between intestinal flora and metabolites at phyla level. *Statistically significant difference (*P* < 0.05). **Statistically significant difference (*P* < 0.01).

In Spearman correlation analysis, different metabolites with the top 20 abundance and family, genus and phylum flora were analyzed to determine the effect of PAL on intestinal flora and metabolic relationships in OA-OP rats. The data showed that at the family level, PAL treatment changed the abundance of Desulfovibrionaceae, Eggerthellaceae, Peptostreptococcaceae, Ruminococcaceae and norank_o_Clostridia_UCG-014 and affected the metabolic levels of 4-ethylphenylsulfate, LysoPC (20:4(5Z, 8Z, 11Z, 14Z)/0:0), 2-hydroxycinnamic acid, Henicosanoylcarnitine, 1-O-hexadecyl-sn-glycero-3-phosphocholine and deoxycytidine (Figure 7A). At the genus level, the abundance changes in Romboutsia, norank_f_norank_o_Clostridia_UCG-014, Desulfovibrio, and Marvinbryantia affected the levels of Henicosanoylcarnitine, 2-hydroxycinnamic acid, 1-O-hexadecyl-sn-glycero-3-phosphate, deoxycytidine and 4-ethylphenylsulfate (Figure 7B). At the phylum level, the abundance changes of Campilobacterota, Cyanobacteria, Desulfobacterota, Firmicutes, and Patescibacteria affected the levels of 1-O-isopentyl-3-O-octadec-2-enoyl glycerol, 4-ethylphenylsulfate, deoxycytidine, P-coumaric acid, 2-hydroxycinnamic acid, (12Z)-10-hydroxy octadecenoyl carnitine, 1-O-hexadecyl-sn-glycero-3-phosphocholine and isenicosanoylcarnitine (Figure 7C).

## 4. Discussion

In this study, we first conducted drug monomer screening based on previous research (19), and then used network pharmacology to further refine the selection. Active compounds in EXD were retrieved and screened from TCMSP and BATMAN-TCM databases. These active compounds were matched with UPLC-QTOF-MS data from previous studies (19), resulting in 17 common active ingredients. We then searched and screened gene targets for OA and OP based on GeneCard, DisGeNET, OMIM, DrugBank, and PharmGKB databases. By conducting topological analysis on the 17 common active ingredients and gene targets of OA-OP, and through analysis of the results and previous literature research, we ultimately chose palmatine for further experiments. For a more detailed description of the selection process for palmatine, please refer to Supplementary File 1.

Network pharmacology, metabolism, and intestinal flora are all interconnected in the human body. The relationship between network pharmacology and metabolism is that drugs can interact with metabolic enzymes, which can affect their pharmacokinetics and pharmacodynamics. Drug may be metabolized by a specific enzyme, and genetic variations in that enzyme may affect how the drug is metabolized, leading to differences in drug efficacy and toxicity. The relationship between metabolism and intestinal flora is that the gut microbiome can also affect drug metabolism. Some gut bacteria can produce enzymes that metabolize drugs, which can affect drug efficacy and toxicity. Additionally, the gut microbiome can affect the absorption of drugs in the intestine, which can also impact drug efficacy and toxicity.

In this study, the effects of PAL on the intestinal flora and serum metabolite profiles of OA-OP rats were determined by non-targeted LC–MS/MS metabonomics and 16S rDNA sequencing techniques. The results showed that PAL treatment could affect the serum metabolite profile and intestinal flora of rats. Metabolites including 5-methoxytryptophol, 2-methoxy acetaminophen sulfate, beta-tyrosine, indole-3-carboxylic acid-O-sulfate and cyclodopa glucoside were upregulated in the KOA group compared to the blank group; however, these metabolites were dialed back after PAL treatment. These metabolites are involved in tyrosine metabolism, phenylalanine metabolism, ubiquinone and other terpenoid-quinone biosynthesis. In terms of the intestinal flora, the abundances of Firmicutes, Bacteroidetes, Actinobacteria, Lactobacillus, unclassified_f_Lachnospiraceae, norank_f_Muribaculaceae, Lactobacillaceae, Lachnospiraceae, and Muribaculaceae were similar to those of the sham group after PAL administration. The results showed that PAL affected the levels of metabolites and the abundance of some intestinal flora in OA-OP rats through multiple targets, thereby improving the physical signs of OA-OP mice.

5-methoxytryptophan (5-MTX) has been found to have potential for radical scavenging and antioxidant activity (27). 5-methotrexate also coordinate the circadian rhythms of a variety of mammals (28). In addition, 5-MTX has important biological functions as an antioxidant, immunomodulator and anticancer agent (29). Interestingly, a study indicated that 5-MTX can inhibit osteoclast formation and promote osteoblast differentiation (29). In addition, 5-MTX can inhibit proinflammatory cytokines, reduce the levels of MMP-2 and MMP-9, and improve synovial inflammation in rats (30). 2-Methoxy acetaminophen sulfate is a member of the acetamide group.

Acetamide has long been considered to be related to the levels of glutathione and N-acetylcysteine. N-acetylcysteine can regulate a variety of pathophysiological processes, including oxidative stress, apoptosis, mitochondrial dysfunction, and imbalance of glutamate and dopamine neurotransmitter systems (31). Other studies have shown that 2-methoxy acetaminophen sulfate has an intervention effect on neurodegenerative diseases (such as amyotrophic lateral sclerosis and frontotemporal dementia) (32). Phenylalanine is one of the essential aromatic amino acids of the human body and can only be obtained from the outside world by food, because there is no relevant synthesis pathway in the human body. Phenylalanine is catabolic mainly in the liver and is catalyzed to form tyrosine by phenylalanine hydroxylase (33). Tyrosine is a non-essential amino acid found in humans and other mammals. Tyrosine catabolism is catalyzed by a variety of metabolic enzymes, including tyrosine aminotransferase, 4-hydroxyphenylpyruvic acid, 4-hydroxyphenylpyruvate dioxygenase, homogentisic acid, homogentisate 1,2-dioxygenase, fumarylacetoacetase, and fumaric acid (34). Depletion of tyrosine metabolizing enzymes leads to the accumulation of metabolites, which further causes DNA damage, tissue damage, and depletion of intracellular glutathione, eventually leading to apoptosis (35). In addition, excessive accumulation of tyrosine in the body can also cause changes in the functions of several key enzymes in the TCA cycle, such as citrate synthase, malate dehydrogenase and succinate dehydrogenase, resulting in the disorder of energy metabolism in the body and the oxidative stress of mitochondria (36). Our results suggest that PAL can improve disease progression in OA-OP rats by regulating tyrosine and phenylalanine metabolism.

A growing number of studies have shown that an imbalance in intestinal homeostasis may induce several extrenteral immune and metabolic diseases (such as osteoporosis, OA, psoriasis, and systemic lupus erythematosus) (37–39). The intestinal flora is a key factor in activating and maintaining intestinal physiological functions and plays an indelible role in maintaining the health and homeostasis of the host (7). In recent years, an increasing number of human and animal studies have indicated the presence of the gut axis and recognized that the gut joint axis and gastrointestinal microbiome-induced immune and inflammatory responses play an important role in joint health (40). It has been shown that the normal human gut microbiome consists of two main phyla, Bacteroidetes and Firmicutes (41). Bacteroides play a role in bone protection by promoting osteoblast differentiation and inhibiting osteoclast differentiation (42, 43). In our results, Bacteroidetes and Firmicutes were downregulated in the OA-OP group compared with the sham group; however, this situation was restored after PAL administration. Lactobacillus is thought to be a beneficial bacterium that potentially affects immune-related bone health by regulating proinflammatory cytokines and markers related to bone metabolism (44). Muribaculaceae belongs to the phylum Bacteroides and was renamed by Ilias et al. Studies have shown that intermittent parathyroid hormone (PTH) can increase the abundance of Muribaculaceae and increase bone mass in OVX rats (45). An epidemiological analysis showed that Lachnospiraceae abundance was reduced in populations with low bone mineral density (BMD). Our study showed that PAL use increased the abundance of Actinobacteriota, Lactobacillus, Lachnospiraceae and Muribaculaceae in OA-OP rats.

Our results demonstrate the potential of PAL to improve OA and OP dual models in rats, where a variety of gut microbes and metabolites may play a role in the recovery mechanism. However, the study has some limitations, including a limited sample size and identified bacteria and metabolites that could not be described as biomarkers in OA-OP rats. Therefore, the results of this study can only provide some references for exploring the mechanism of bone mass loss and cartilage degeneration in rats with osteoporosis and osteoarthritis inflammation, which needs to be verified in a large sample size study. Further, in animal studies, we will further add different dosing concentrations and positive control drugs in the future to improve the reliability of our conclusions. In addition, we will explore the impact of GM and metabolites on OA-OP through additional experiments in the future.

## 5. Conclusion

In conclusion, PAL can improve cartilage degeneration and bone mass loss in OA-OP rats. The potential mechanism of action may be related to the improvement of intestinal microbiome ecological imbalance and to balance of phenylalanine/tyrosine metabolism disorder. Moreover, there is a potential role of the GM as a shared mechanism for two common age-related diseases. In addition, the key genera of the GM detected in this study may help identify potential therapeutic targets for joint degradation and bone mass loss in the GM.

## Data availability statement

The data presented in this study are deposited in the NCBI repository, accession number PRJNA929116.

## Ethics statement

This animal study was reviewed and approved by the Animal Ethics Committee of Nanjing University of Chinese Medicine.

## Author contributions

LJ and ZM conceived the study, designed the experiments, participated in the literature searched, and extracted the data. PW and JM participated in study design, drafted the manuscript, devised the study, and oversaw the research program. XS, YG, and LY participated in data analysis, performed the statistical analysis, and drafted the manuscript. All authors read and approved the final manuscript.

## Footnotes

1
https://old.tcmsp-e.com/tcmsp.php

2
http://bionet.ncpsb.org.cn/batman-tcm/

3
https://www.genecards.org/

4
https://www.disgenet.org/

5
https://omim.org/

6
https://go.drugbank.com/

7
https://www.pharmgkb.org/

## Supplementary material

Supplementary Table 1 Topological analysis based on network pharmacology.

Supplementary Table 2 Potential metabolites.

Supplementary Table 3 Potential metabolic pathways.

Supplementary File 1 The flow chart of a detailed procedure how palmatine was identified.
